# Effect of Sintering Conditions and Acidic Environments on the Wear of 5Y-ZP Zirconia

**DOI:** 10.3390/dj14080527

**Published:** 2026-08-18

**Authors:** Benya Kangsanarak, Wissanee Jia-Mahasap, Pimduen Rungsiyakull

**Affiliations:** 1 Department of Prosthodontics, Faculty of Dentistry, Chiang Mai University, Chiang Mai 50200, Thailand; benya_kan@cmu.ac.th; 2 Department of Restorative Dentistry and Biomaterials Sciences, Harvard School of Dental Medicine, Boston, MA 02115, USA; wissanee_jia-mahasap@hsdm.harvard.edu

**Keywords:** dental, zirconia, speed sintering, acidic environments, wear resistance

## Abstract

**Background/Objectives**: This study evaluated the influence of sintering conditions and acidic environments on the wear of 5 mol% yttria-stabilized zirconia (5Y-ZP). **Methods**: Sixty zirconia specimens were divided into two sintering groups (conventional and speed) and three immersion media (n = 10): distilled water, Coca-Cola, and vinegar. Wear testing was performed using a chewing simulator under a 49 N load for 120,000 cycles, simulating 6 months of clinical function. Volume loss, mean wear depth, and surface roughness were measured using a 3D profilometer. Surface and compositional analyses were performed using scanning electron microscopy (SEM), energy-dispersive X-ray spectroscopy (EDS), and X-ray diffraction (XRD). **Results**: Significant differences in wear were observed (*p* < 0.05). The greatest wear occurred in distilled water (58.927 ± 13.199 µm; 0.3518 ± 0.1062 mm^3^ for speed-sintered and 53.408 ± 6.784 µm; 0.3211 ± 0.0546 mm^3^ for conventional). Vinegar produced moderate wear (15.478 ± 2.584 µm; 0.0482 ± 0.0142 mm^3^ and 11.575 ± 3.334 µm; 0.0360 ± 0.0116 mm^3^), whereas Coca-Cola produced the least (2.899 ± 1.377 µm; 0.0049 ± 0.0027 mm^3^ and 1.870 ± 0.688 µm; 0.0027 ± 0.0012 mm^3^). Sintering condition affected only wear depth. SEM showed localized surface alterations in acidic media, EDS revealed reduced yttria content, and XRD revealed no detectable monoclinic phase, with no interaction between variables. **Conclusions**: Acidic environments had a greater influence on the wear of 5Y-ZP zirconia than sintering conditions. Speed sintering influenced wear far less than the immersion medium, supporting its use in patients exposed to acidic conditions.

## 1. Introduction

Yttria-stabilized tetragonal zirconia polycrystal (Y-TZP) is widely used in restorative dentistry due to its excellent mechanical properties [1], biocompatibility [2], and high wear resistance [3]. However, the original 3 mol% yttria-stabilized tetragonal zirconia polycrystal (3Y-TZP) exhibited limited esthetic performance due to its opacity [4]. To address this limitation, a more translucent 5 mol% yttria-stabilized zirconia (5Y-ZP) was developed by increasing the yttria content, reducing alumina, enhancing material density, and increasing the proportion of the cubic phase, all contributing to enhanced optical properties [5,6,7]. These modifications, however, reduce phase transformation toughening [8], resulting in lower flexural strength and fracture toughness compared to 3Y-TZP. Although 5Y-ZP zirconia differs in microstructure from 3Y-TZP, several studies have indicated that such variations do not substantially affect the wear resistance of these materials [9,10].

Among its many favorable properties, zirconia is particularly recognized for its wear resistance, which is attributed to its dense microstructure and high fracture toughness. Ideally, restorative materials should mimic the wear characteristics of natural enamel to maintain functional harmony. Natural enamel typically exhibits a wear rate of approximately 30–40 µm per year in the molar region [11,12]. The wear resistance of zirconia is influenced by multiple factors, including its microstructure, surface roughness, and the chemical environment in the oral cavity [13]. Among these, surface roughness plays a critical role [12]. Smoother zirconia surfaces tend to produce less wear on opposing teeth [14], whereas rougher surfaces can lead to increased friction and contribute to material loss. Normally, zirconia undergoes two-body wear, involving direct contact between surfaces. However, microfractures during function can generate debris, which becomes entrapped between contacting surfaces, resulting in three-body wear and accelerated material loss [15].

In addition to mechanical factors, chemical conditions within the oral environment, particularly exposure to acidic substances, can significantly influence the surface properties. Dental erosion, a progressive loss of tooth structure caused by nonbacterial acids [16,17], may result from intrinsic sources such as bulimia or gastroesophageal reflux disease (GERD), both involving repeated exposure to gastric contents, or extrinsic sources like acidic foods and beverages [18,19]. This condition may lead to sensitivity, esthetic concerns, and loss of vertical dimension [20,21]. The erosive potential of beverages depends on pH, acid type, temperature, concentration, and exposure time [22]. Common acids include phosphoric acid in cola beverages, citric acid in citrus juices, and acetic acid in vinegar-based products [23]. While various factors influence erosivity, the simulation of acid exposure remains inconclusive. Previous studies have suggested that immersion in acid at pH 1.2 for 1 h may correspond to approximately 1.25 to 4 months of clinical exposure [19,24,25,26]. Although zirconia is known for its chemical stability [19], prolonged exposure to strong acids may cause surface degradation, yttria loss [27], and phase transformation, which can adversely affect its mechanical properties [28,29]. Understanding these effects is essential for selecting suitable restorative materials, especially for patients at high risk of dental erosion.

Despite its advantages, one significant limitation of zirconia is its traditionally long sintering time, which typically requires 6 to 8 h [30]. This extended sintering duration can be inefficient for clinical applications and dental laboratory operations. To address this issue, speed-sintering conditions have been developed, allowing zirconia restorations to be fabricated while significantly reducing the sintering time to approximately 30 min. This method offers significant time-saving potential, particularly for chairside and same-day dentistry applications. However, the effects of altered sintering conditions on zirconia properties remain controversial, especially regarding wear resistance.

Previous studies on speed-sintered zirconia have reported inconsistent outcomes, ranging from similar [31,32] to inferior [33,34] or superior [8,35,36] wear resistance compared to conventionally sintered zirconia. However, limited research has specifically investigated the wear behavior of 5Y-ZP zirconia, particularly under acidic environments. To date, no study has comprehensively examined the combined effects of sintering conditions and acidic environments on the wear resistance of 5Y-ZP zirconia.

Therefore, the aim of this study was to investigate the effects of different sintering conditions and acidic environments on the wear resistance of 5Y-ZP zirconia. The null hypothesis was that the sintering conditions and acidic environments would not significantly influence the wear resistance of 5Y-ZP zirconia.

## 2. Materials and Methods

A minimum sample size of 40 was calculated using G*Power software (version 3.1.9.7; Heinrich-Heine-Universität Düsseldorf, Düsseldorf, Germany) [37], with a statistical power of 80%, a significance level of α = 0.05, and an effect size (f) = 0.52. To compensate for potential specimen loss, the number was increased to a final total of 60 specimens (n = 10 per group).

### 2.1. Specimen Preparation

Sixty flat zirconia specimens were prepared by milling 5Y-ZP zirconia discs (KATANA STML zirconia; Kuraray Noritake Dental Inc., Tokyo, Japan). To optimize material usage, the specimens were milled along the disc diameter, starting with a length of 70 mm and gradually shortened to 68 mm, 60 mm, and 45 mm depending on their position from the center, while maintaining a constant width and height of 6 mm. The zirconia used in this study was multilayered, consisting of an enamel-like translucent layer, a dentin layer, and a transitional layer. The enamel side was selected for testing, as it was designed by the manufacturer to serve as the occlusal surface of restorations in clinical applications. To ensure proper specimen orientation, a groove was milled on the opposite side, corresponding to the dentin layer. This marking clearly identified the dentin side and prevented confusion during specimen preparation and testing.

The specimens were then sectioned into 6 mm thick slices using a precision saw (Isomet 1000; Buehler, Lake Bluff, IL, USA) to obtain a final dimension of 6 × 6 × 6 mm^3^ after sintering. The specimens were thoroughly cleaned to remove milling residue and sintered according to the manufacturer’s instructions. Thirty specimens were sintered in a furnace (inLab Profire; Dentsply Sirona, Bensheim, Germany) using the conventional sintering protocol, which followed a 6 h and 58 min thermal cycle including a 2 h holding time at 1550 °C. The remaining thirty specimens underwent speed sintering in the same furnace, following a 53 min thermal cycle with a 20 min holding time at 1600 °C.

After sintering, the specimens were embedded in a circular aluminum metal holder and secured in place with epoxy resin (Atfiber Corp., Chon Buri, Thailand). During this step, special attention was given to positioning the dentin side with the milled groove facing downward and the enamel side facing upward, ensuring it remained parallel to the horizontal plane to facilitate consistent wear testing. The surfaces of all flat specimens were polished with 320, 600, and 1200 grit silicon carbide papers under water cooling using a rotary polisher (LaboPol-20, Struers, Copenhagen, Denmark) to obtain a flat surface parallel to the horizontal plane before testing. Prior to wear testing, all specimens were immersed in distilled water at room temperature for 7 days as a standardized hydration step applied equally to every group, regardless of the immersion medium subsequently assigned.

The specimens were divided into six groups (n = 10 per group) according to the sintering conditions and immersion medium. Within each sintering group, the specimens were further divided into three subgroups, with each subgroup immersed in one of the following solutions: distilled water, bottled Coca-Cola Classic (The Coca-Cola Company, Bangkok, Thailand), or distilled vinegar containing 5% (*w*/*v*) acetic acid (PFO Food, Bangkok, Thailand). Coca-Cola and distilled vinegar were selected as representative acidic solutions to simulate the conditions of daily dietary exposure. The pH values of the testing solutions were measured and recorded at room temperature using a pH meter (Mettler Toledo, Five Easy F20, Greifensee, Switzerland). The measured pH values were 2.5 for Coca-Cola, 2.6 for vinegar, and 7.0 for distilled water. The six resulting groups were labeled as follows: conventional sintering in water (CW), Coca-Cola (CC), and vinegar (CV); and speed sintering in water (SW), Coca-Cola (SC), and vinegar (SV). Antagonists were fabricated using 5Y-ZP zirconia discs (KATANA STML zirconia; Kuraray Noritake Dental Inc., Tokyo, Japan) to provide a standardized surface, as human enamel may vary in morphology and mechanical properties. The discs were milled into hemispherical shapes to simulate the morphology of a molar cusp [38,39], with final dimensions of 4 mm in diameter and 8 mm in height after sintering. These specimens were sintered following the manufacturer’s conventional protocol and subsequently embedded in aluminum holders, where they were fixed with epoxy resin, leaving approximately 2 mm of the hemispherical tip exposed. Polishing was performed using a two-step zirconia polishing kit (Komet; Gebr. Brasseler GmbH & Co. KG, Lemgo, Germany) attached to a low-speed handpiece (Handy 700; Marathon, Saeyang Microtech, Daegu, Republic of Korea) at 6000 rpm under water cooling [40]. Each polishing step was conducted for 30 s in one direction, followed by another 30 s in the perpendicular direction. Baseline surface roughness (R_a_) was recorded using a contact profilometer (Surftest SJ-310; Mitutoyo, Kanagawa, Japan). Each specimen was measured three times with a 2 µm tip radius stylus. For flat specimens, a 1.5 mm evaluation length with a 0.25 mm sampling length was used; for hemispherical specimens, the values were 0.48 mm and 0.08 mm, respectively. Measurements were conducted using a 2.5 µm cutoff wavelength and a scanning speed of 0.5 mm/s. A contact profilometer was preferred over an optical profilometer to minimize measurement errors caused by light reflection from the highly polished zirconia surfaces. Moreover, the high hardness of fully sintered zirconia minimized the risk of surface scratching or deformation during scanning. All specimens were polished to achieve baseline R_a_ values below 0.2 µm [5]. No significant difference in surface roughness was found among groups based on ANOVA analysis (*p* > 0.05).

### 2.2. Wear Simulation Test

**Figure 1:**
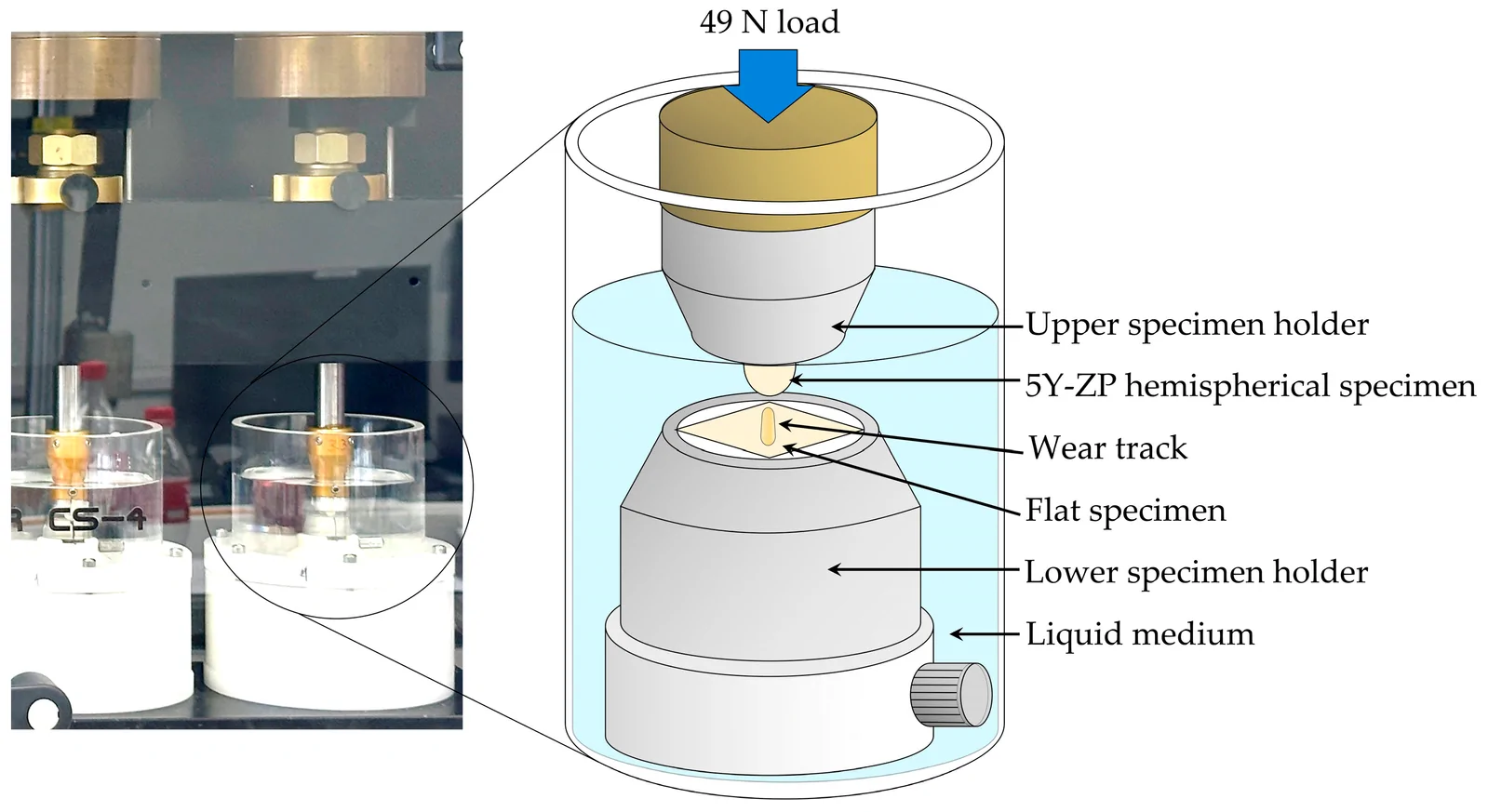
Chewing simulator setup for wear testing. (**Left**) The chewing simulator (CS-4.4; SD Mechatronik) used in this study. The circled area indicates the contact zone, shown as a detailed schematic (**right**). The diagram illustrates the 5Y-ZP hemispherical antagonist, the flat zirconia specimen mounted in an aluminum specimen holder, the wear track, and the liquid medium chamber. A constant load of 49 N was applied during the testing cycles.

Wear testing was conducted using a dual-axis chewing simulator (Chewing Simulator CS 4.4; SD Mechatronik, Feldkirchen-Westerham, Germany). The flat specimen was positioned in the lower specimen holder, and a hemispherical zirconia antagonist was mounted in the upper chamber, aligned above the center of each specimen. A load of 49 N was applied until passive contact was established, which is comparable to the average human chewing force [41]. The antagonists moved horizontally in a 2 mm range at 1.64 Hz for 120,000 cycles, simulating approximately 6 months of oral function [42]. During the entire 120,000-cycle test, each specimen remained fully submerged in its assigned immersion medium (distilled water, Coca-Cola Classic, or vinegar) within a static liquid chamber at room temperature. Specimens were not pre-soaked in their acidic medium; acidic exposure began at the start of wear testing. After testing, specimens were ultrasonically cleaned in distilled water for 5 min, air-dried, and stored at room temperature. The chewing simulator setup used for wear testing is shown in Figure 1.

### 2.3. Quantitative Wear Analysis

After testing, worn flat specimens were scanned using a 3D profilometer (Talyscan 150; Taylor Hobson SA, Leicester, UK) using step sizes of 0.5 µm (X-axis) and 20 µm (Y-axis) with a 60 nm resolution and 1 mm/s speed. According to the manufacturer, the Talyscan 150 provides a lateral resolution of 0.5 µm (X-axis), 5 µm (Y-axis), and a vertical (Z-axis) resolution of 60 nm, allowing high-precision three-dimensional wear volume measurements. The volume loss (mm^3^) and mean wear depth (µm) were calculated using software (TalyMap Universal, Taylor Hobson SA, Leicester, UK), referencing the unworn surface as the baseline. Surface roughness (R_a_) was measured at both the center of the wear track and the adjacent unworn area of each specimen, using a sampling length of 1.5 mm, a cutoff wavelength of 25 µm, and a measurement speed of 1 mm/s. All quantitative wear measurements were performed by a single, non-blinded operator using standardized instrument settings, without repeated measurements.

### 2.4. Qualitative Wear Analysis

#### 2.4.1. Scanning Electron Microscopy (SEM) and Energy-Dispersive X-Ray Spectroscopy (EDS) Analysis

The flat zirconia specimens were coated with a thin layer of gold using a sputter coater (Cressington Sputter Coater 6006; Cressington Scientific Instruments Ltd., Watford, UK) to enhance surface conductivity for qualitative wear analysis. SEM imaging was performed using a scanning electron microscope (Tescan Vega 4; Tescan Orsay Holding, Brno, Czech Republic) at an accelerating voltage of 15 kV, with a working distance of approximately 14–15 mm, and magnifications ranging from 180× to 50,000×. Surface elemental composition was analyzed using energy-dispersive X-ray spectroscopy (EDS) with a detector (Xplore 30; Oxford Instruments, High Wycombe, UK) integrated with the SEM. Data were collected and analyzed using the software program (Aztec version 6.1; Oxford Instruments, High Wycombe, UK). EDS analysis was performed at a central location within each wear track using an accelerating voltage of 15 kV and a magnification of 10,000×. The selected area included both rough and smooth surface features. EDS measurements were taken at two points on each surface type. The identified elements were reported as atomic percentage (at.%) and weight percentage (wt.%).

#### 2.4.2. Phase Analysis

The crystalline phase structure of representative zirconia specimens from each group was evaluated using an X-ray diffractometer (XRD; SmartLab, Rigaku, Tokyo, Japan). Measurements were performed using Cu Kα radiation at 40 kV and 40 mA over a 2θ range of 20–90° with a step size of 0.02°. The specimens remained embedded in circular aluminum metal holders during measurement, as removal was not feasible without damaging the samples. To assess potential interference from the holder material, the aluminum holder alone was also analyzed under identical conditions. Additionally, a conventionally sintered control specimen without wear testing was included as a baseline reference for phase comparison.

### 2.5. Statistical Analysis

Statistical analyses were performed using a statistical software program (IBM SPSS Statistics, version 26; IBM Corp., Armonk, NY, USA). The normality of data distribution was assessed using the Shapiro–Wilk test, and all groups showed normal distribution (*p* > 0.05). Therefore, a two-way analysis of variance (ANOVA) was applied as a parametric test to evaluate the main effects of sintering conditions and acidic environments, as well as their interaction, on wear outcomes. Homogeneity of variance was examined using Levene’s test. Since heterogeneous variances were detected (*p* < 0.05), post hoc multiple comparisons were performed using Dunnett’s T3 test. The significance level was set at *p* < 0.05.

## 3. Results

### 3.1. Quantitative Wear Outcomes

**Figure 2:**
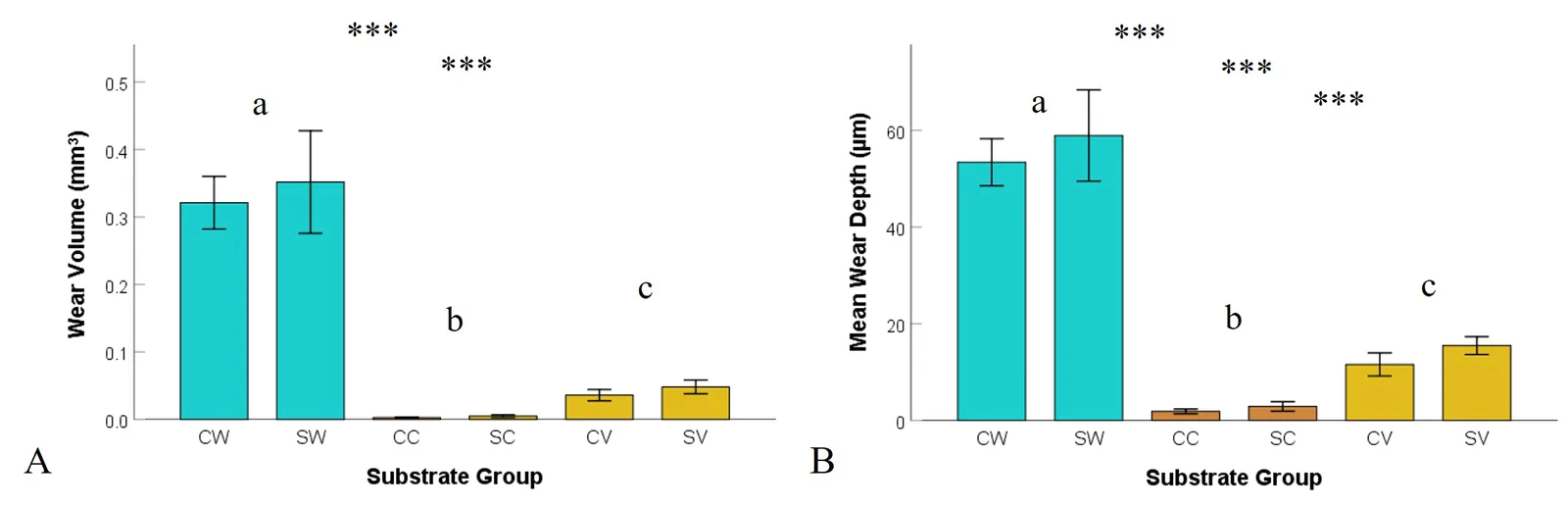
Wear outcomes of 5Y-ZP zirconia specimens after 120,000 cycles of chewing simulation. (**A**) Mean wear volume loss (mm^3^). (**B**) Mean vertical wear depth (µm) measured using 3D profilometry. Bars represent mean ± standard deviation. Colors indicate different environments. Groups sharing the same lowercase letter (a–c) are not significantly different (*p* > 0.05). Asterisks (***) indicate significant differences between immersion environments (*** *p* < 0.001). Specimen codes represent sintering condition and immersion environment: C = conventional sintering, S = speed sintering; W = distilled water, C = Coca-Cola, and V = vinegar.

**Figure 3:**
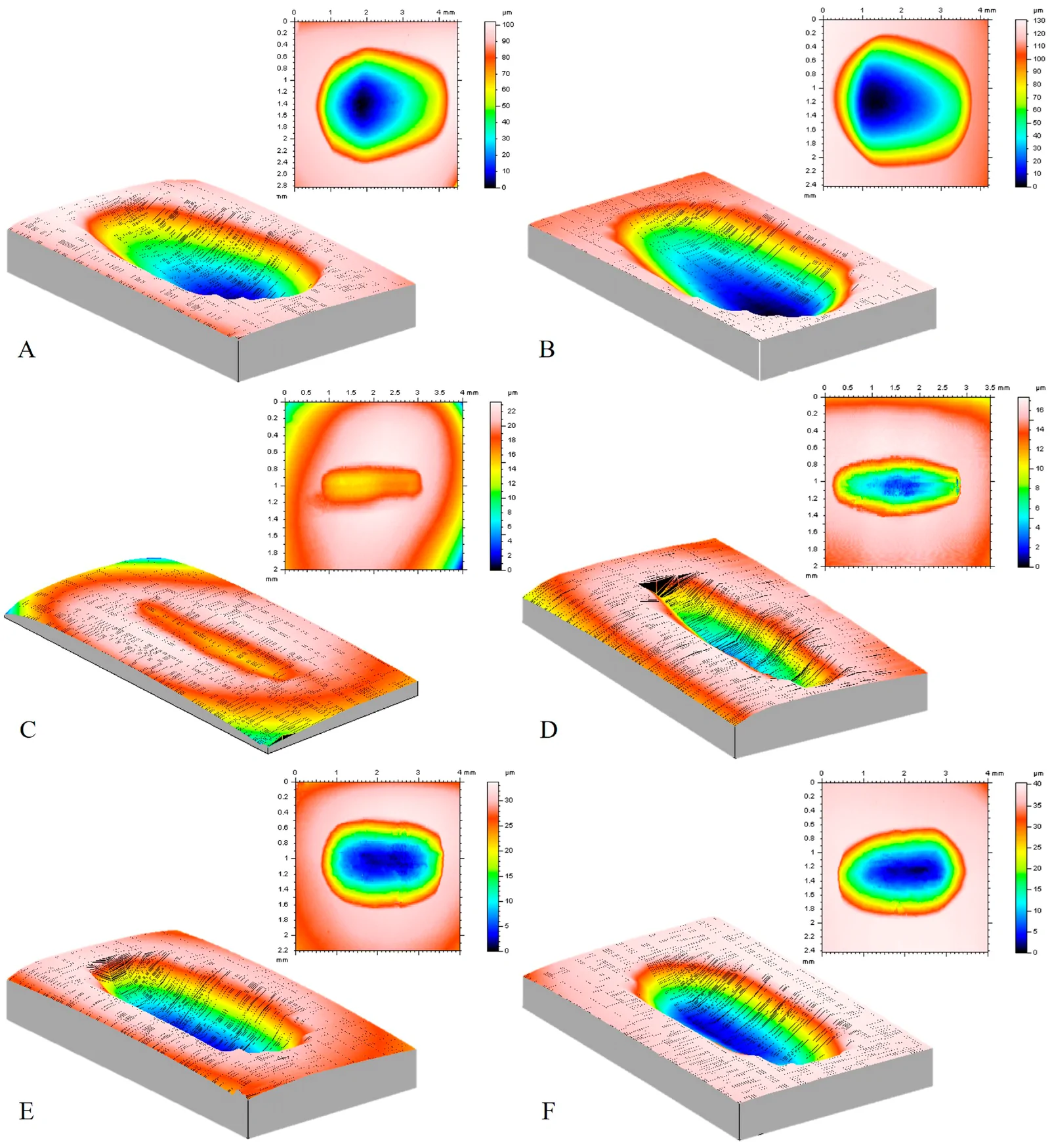
Three-dimensional scans of wear tracks of representative flat specimens after 120,000 wear cycles. (**A**) Conventionally sintered zirconia in water. (**B**) Speed-sintered zirconia in water. (**C**) Conventionally sintered zirconia in Coca-Cola. (**D**) Speed-sintered zirconia in Coca-Cola. (**E**) Conventionally sintered zirconia in vinegar. (**F**) Speed-sintered zirconia in vinegar.

The volume loss (mm^3^) and mean wear depth (µm) for each group, as measured with a 3D profilometer after 120,000 wear cycles, are presented in Table 1 and Figure 2, while the 3D wear characteristics of representative specimens are shown in Figure 3.

The highest mean wear depth and volume loss were observed in the SW group (58.927 ± 13.199 µm, 0.3518 ± 0.1062 mm^3^), which was not significantly different from the CW group (53.408 ± 6.784 µm, 0.3211 ± 0.0546 mm^3^) (*p* = 0.732). Intermediate values were recorded in the SV group (15.478 ± 2.584 µm, 0.0482 ± 0.0142 mm^3^) and the CV group (11.575 ± 3.334 µm, 0.0360 ± 0.0116 mm^3^) (*p* = 1.000). The lowest values were found in the SC group (2.899 ± 1.377 µm, 0.0049 ± 0.0027 mm^3^) and CC group (1.870 ± 0.688 µm, 0.0027 ± 0.0012 mm^3^) (*p* = 0.993), with no significant differences between corresponding pairs.

### 3.2. Surface Roughness

Surface roughness measured at the center of the wear track is presented in Table 1. The highest surface roughness was recorded in the CW group (0.053 ± 0.012 µm), which was not significantly different from the SW (0.052 ± 0.007 µm) (*p* = 1.000), SV (0.042 ± 0.011 µm) (*p* = 0.141), and CV (0.040 ± 0.011 µm) groups (*p* = 0.058). However, both CW and SW exhibited significantly higher roughness than the CC (0.033 ± 0.006 µm) (*p* < 0.001) and SC (0.034 ± 0.010 µm) (*p* = 0.001) groups. No significant differences were found between the SV and CV groups and the SC and CC groups.

Two-way ANOVA results are summarized in Table 2. The immersion medium was the dominant factor, significantly affecting volume loss, mean wear depth, and surface roughness (*p* < 0.001) with large effect sizes (partial η^2^ = 0.42–0.94). In contrast, the sintering condition had no significant effect on volume loss (*p* = 0.243) or surface roughness (*p* = 0.717). For mean wear depth, a significant but comparatively small effect was found (*p* = 0.038, partial η^2^ = 0.078), substantially smaller than the effect of the immersion medium on the same outcome (partial η^2^ = 0.937). No significant interaction was found for any outcome (*p* = 0.529–0.925). Post hoc comparisons showed no significant difference between the two sintering conditions within any individual immersion medium (Section 3.1). Among the three media, Coca-Cola caused the least wear and distilled water the greatest.

### 3.3. Surface Morphology and Elemental Composition

**Figure 4:**
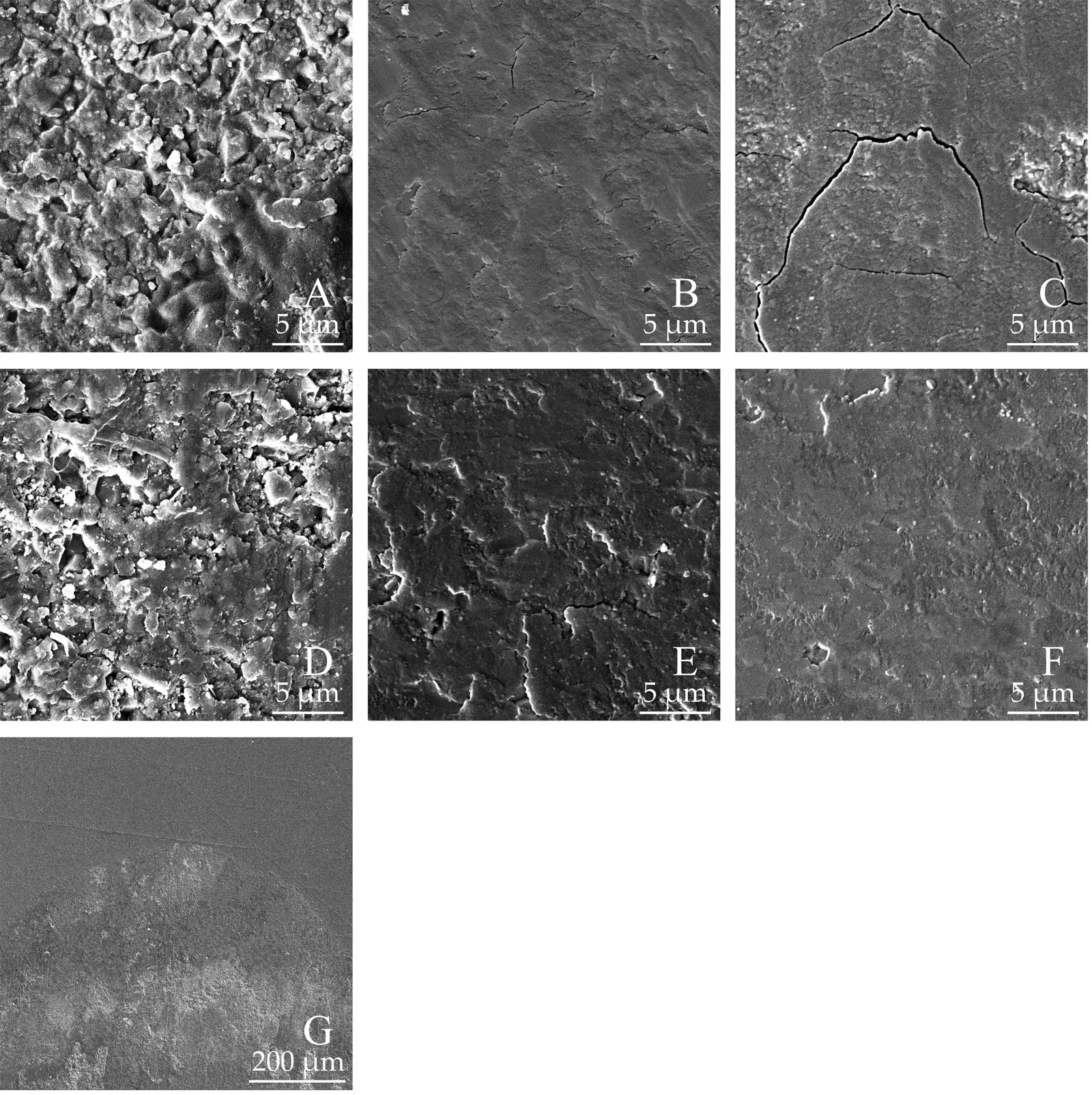
Scanning electron microscope images of zirconia specimens after wear testing. Panels (**A**–**F**) show the center of the wear track, acquired at an accelerating voltage of 15 kV, a working distance of approximately 14–15 mm, and a magnification of 30,000×, illustrating surface morphology and wear features under different immersion conditions. (**A**) Conventionally sintered zirconia in distilled water. (**B**) Conventionally sintered zirconia in Coca-Cola. (**C**) Conventionally sintered zirconia in vinegar. (**D**) Speed-sintered zirconia in distilled water. (**E**) Speed-sintered zirconia in Coca-Cola. (**F**) Speed-sintered zirconia in vinegar. (**G**) Representative image (1000×) of a speed-sintered specimen immersed in vinegar, showing the boundary between the wear track and the adjacent unworn region.

**Figure 5:**
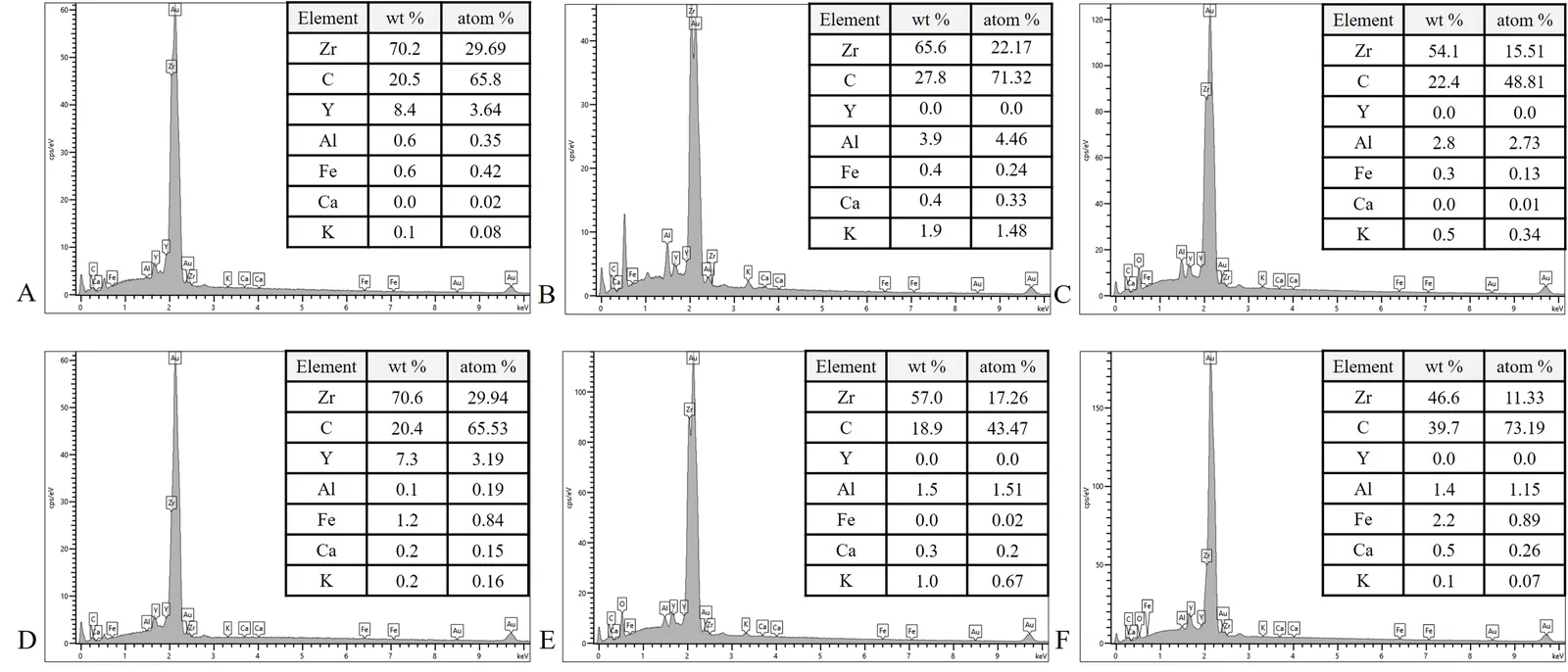
Energy-dispersive X-ray spectroscopy (EDS) spectra and corresponding elemental composition tables (wt.% and at.%) of representative zirconia specimens after wear testing, showing the surface elemental composition under different immersion conditions. (**A**) Conventionally sintered zirconia in distilled water. (**B**) Conventionally sintered zirconia in Coca-Cola. (**C**) Conventionally sintered zirconia in vinegar. (**D**) Speed-sintered zirconia in distilled water. (**E**) Speed-sintered zirconia in Coca-Cola. (**F**) Speed-sintered zirconia in vinegar.

**Figure 6:**
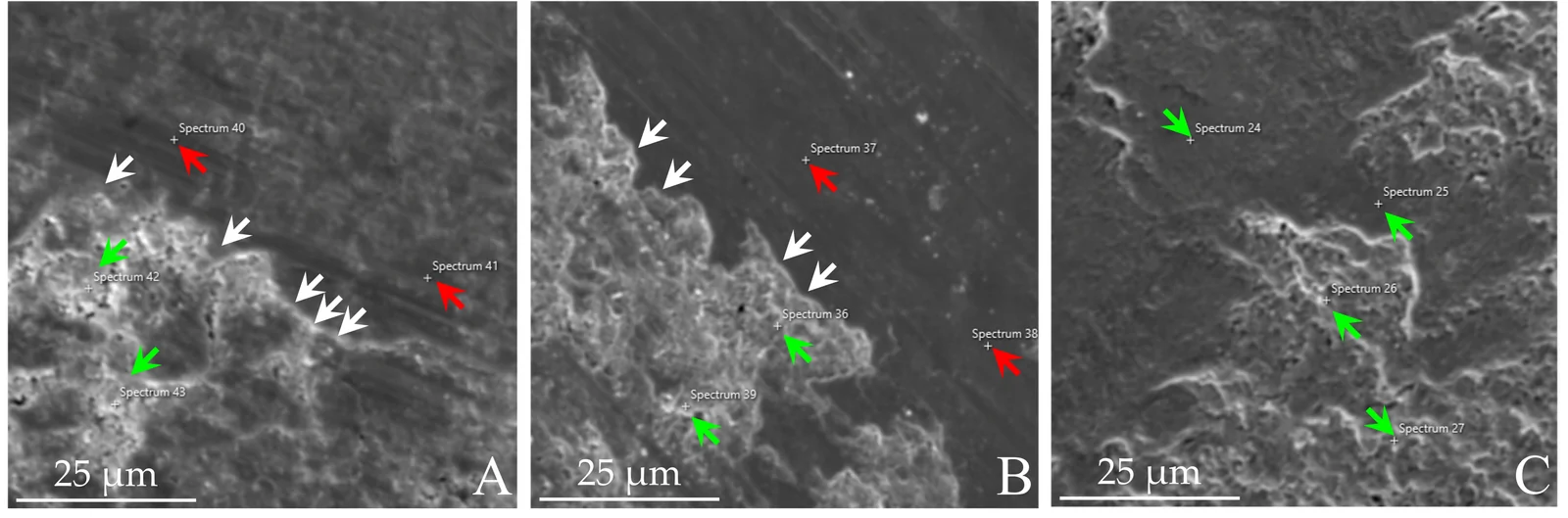
Representative SEM images with EDS analysis demonstrating the microstructural characteristics of zirconia specimens after wear testing. (**A**,**B**) Specimens exposed to acidic conditions reveal distinct microstructural alterations. The red arrow indicates areas with a lower measured yttrium signal, the green arrow indicates a smaller reduction in the measured yttrium signal, and the white arrows highlight the interfaces between zirconia regions with different microstructural features. (**C**) The specimen stored in distilled water shows the presence of yttrium with only minor reduction, suggesting less pronounced microstructural changes compared with acidic conditions. EDS is semi-quantitative and surface-sensitive; these observations reflect localized surface signal differences rather than confirmed bulk compositional change.

SEM imaging revealed distinct microstructural differences among the specimens immersed in different pH solutions. A representative image showing the boundary between the worn and adjacent unworn regions is presented in Figure 4G, allowing direct comparison of the surface morphology within and outside the wear track on the same specimen. The wear tracks of distilled water-immersed specimens exhibited more surface irregularities with uneven dark and white areas, compared to those in vinegar and Coca-Cola. Clusters of micropores and microcracks were observed at 30,000× magnification (Figure 4B,C,E) in the CC, CV, and SC groups. Additionally, the grain boundaries of vinegar-immersed specimens appeared less distinct than in the other groups. The surface elemental composition (at.%) determined by EDS analysis is summarized in Table 3, revealing the presence of Zr, C, Y, Al, and trace amounts of Fe, Ca, and K. Figure 5 presents the EDS spectra of representative specimens from each group, illustrating the elemental composition of the worn zirconia. A lower Y content was observed in specimens immersed in vinegar and Coca-Cola, approaching very low levels, compared to those in distilled water. Furthermore, SEM imaging revealed distinct microstructural variations within the same wear track region, as shown in Figure 6. EDS analysis further indicated variations in Y content. The smoother areas showed a markedly lower measured Y signal (approaching the detection threshold), whereas the porous regions exhibited a reduction in Y content compared to those immersed in distilled water, but a smaller reduction in measured Y signal.

### 3.4. Phase Analysis

**Figure 7:**
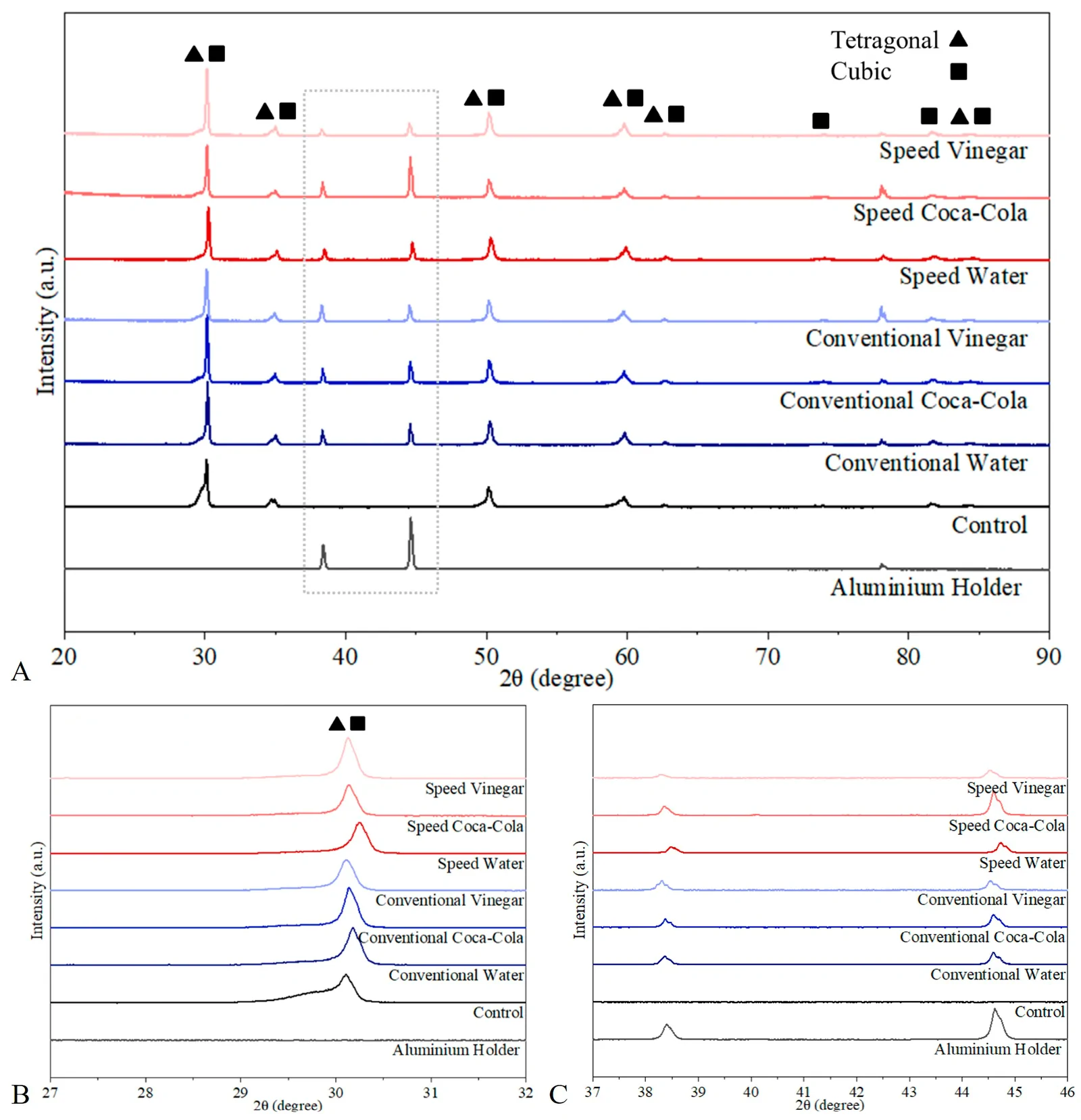
XRD patterns of representative zirconia specimens from each group after wear testing. (**A**) Full diffraction patterns recorded in the 20–90° (2θ) range; the dashed box highlights the region corresponding to additional diffraction peaks at approximately 38.5° and 44.7°. (**B**) Enlarged view of the 27–32° (2θ) region. (**C**) Enlarged view of the 37–46° (2θ) region. Characteristic peaks of cubic and tetragonal zirconia are indexed. The diffraction peaks at approximately 38.5° and 44.7° correspond to the aluminum holder material and were not observed in the unworn control specimen.

The XRD patterns of all specimens showed similar diffraction profiles (Figure 7A), with a prominent peak at approximately 30°, corresponding to the tetragonal phase of zirconia. No detectable peaks were observed at approximately 28° or 31°, the characteristic positions of the monoclinic phase. No new peaks or peak splitting were observed among the different immersion groups. A slight shift toward higher 2θ values was observed in the SW specimen. The unworn control specimen exhibited a diffraction pattern comparable to those of the tested groups. Additional diffraction peaks at approximately 38.5° and 44.7° were observed only in specimens measured within the aluminum holders and were not detected in the control specimen. As shown in Figure 7C, these reflections coincide with the diffraction peaks of the aluminum holder.

## 4. Discussion

This study demonstrated statistically significant differences in wear depth, volume loss, and surface roughness among the experimental groups, particularly in response to varying acidic environments. Therefore, the null hypothesis was rejected for both factors. The immersion medium significantly influenced volume loss, mean wear depth, and surface roughness, whereas the sintering condition significantly influenced only the mean wear depth. The acidic environments had a greater influence on the wear behavior of 5Y-ZP zirconia than the sintering conditions. Furthermore, no significant interaction was observed between the acidic environment and the sintering condition, indicating that their effects were independent.

After 120,000 cycles, which simulate approximately 6 months of clinical use, the mean wear depth of zirconia in distilled water was 53.408 µm for conventional and 58.927 µm for speed sintering, corresponding to approximately 107 and 118 µm per year if wear is assumed to progress linearly, thus exceeding both the recommended limit of 50 µm per year for dental materials [42] and the 30–40 µm per year reported for physiological enamel wear in the molar region [11,12]. In contrast, the corresponding annualized values in vinegar (23–31 µm) and Coca-Cola (4–6 µm) remained below the 50 µm per year limit and were within or below the range reported for physiological enamel wear. The overall wear depth in this study was greater than the values reported in previous studies [8,40,43], which may be attributed to differences in antagonist materials, number of cycles, and the potential involvement of a three-body wear mechanism. The annualized values should be interpreted with caution. The 50 µm per year limit [42] applies to dental restorative materials in general and not specifically to monolithic zirconia, and the wear of the antagonist was not measured in this study. The present findings therefore indicate that neither acidic medium accelerated the wear of 5Y-ZP zirconia. Because restoration longevity also depends on fatigue and fracture behavior, which were not assessed, these results describe wear performance rather than restoration survival.

The greater wear of zirconia observed in distilled water after chewing simulation may be related to surface electrochemical interactions influenced by the surrounding pH environment. Previous studies have reported that the isoelectric point (IEP) of yttria-stabilized zirconia is close to neutral pH [44], which may influence particle–particle and particle–surface interactions during wear. Under near-neutral conditions, reduced surface charge may promote agglomeration of wear debris and the formation of a tribochemical layer on the contact surface [45]. The repeated formation and fracture of this layer may contribute to increased material loss during sliding contact. These reported IEP values, however, are based primarily on zirconia powders, and the electrochemical behavior of fully sintered zirconia may differ. The precise contribution of surface electrochemical factors such as IEP remains speculative and warrants direct experimental confirmation. As distilled water does not represent saliva, this result should not be interpreted as clinical dietary advice. Nevertheless, exposure to the acidic media did not increase wear, and no monoclinic phase formation was detected, indicating that 5Y-ZP zirconia was not degraded under the acidic conditions examined.

Although Coca-Cola and vinegar had similar pH values, different wear behaviors were observed, suggesting that pH alone may not determine zirconia wear. Differences in acid type and chemical composition, including phosphoric acid in Coca-Cola and acetic acid in vinegar, as well as their buffering capacity, may influence surface reactions and tribochemical interactions. Therefore, the wear behavior of zirconia may depend not only on acidity but also on the nature of the acidic medium.

Surface roughness is a critical factor in esthetics and biofilm resistance [46,47]. Surface roughness influences the wear mechanism of zirconia, with higher surface roughness leading to greater material loss and increased wear on the opposing tooth structure [38,48,49]. In this study, a contact profilometer was used instead of an optical profilometer to improve measurement accuracy by minimizing reflection artifacts. The results showed that acidic conditions significantly reduced surface roughness at the center of the wear track, a finding consistent with previous studies [19,50,51]. Under neutral conditions, increased surface roughness enhances friction between contacting surfaces during wear testing. This elevated friction can increase the temperature at the contact interface, potentially triggering a tetragonal-to-monoclinic phase transformation, which has been reported in 3Y-TZP [45]. However, 5Y-ZP is generally considered more resistant to phase transformation owing to its higher yttria content and predominantly cubic phase structure, and no monoclinic peaks were detected under the present experimental conditions. A previous study of the same zirconia material likewise reported no detectable monoclinic phase after either conventional or speed sintering, although that study did not involve wear testing or acidic exposure [52]. The diffraction patterns of the control and tested specimens were comparable, with no additional peaks indicating phase transformation. A slight shift toward higher 2θ values was observed in the SW group; such shifts have been associated with lattice strain or internal stress in previous studies [53], but given the small magnitude of the shift and the absence of direct stress measurements in this study, this finding should be interpreted with caution, and no clear conclusion can be drawn regarding its relationship with wear behavior. The diffraction peaks detected in the 37–46° region were attributed to the aluminum holder rather than to structural changes in zirconia, based on comparison with the reference pattern of the holder (Figure 7C). Although no monoclinic phase was detected within the resolution of the present measurements, future studies incorporating quantitative phase analysis, such as Rietveld refinement, may provide a more precise evaluation of phase fractions, since qualitative XRD analysis may not reliably distinguish between phases with overlapping peak positions, such as rhombohedral and monoclinic phases. Surface roughness at the center of the wear track remained below 0.2 µm, an acceptable threshold for dental materials [54].

Notably, no visible alteration of the surface adjacent to the wear track was observed in the specimen shown in Figure 4G. Together with the absence of a detectable monoclinic phase, this suggests that the observed alterations were confined to the wear track. Therefore, the observed degradation was localized within the wear track and primarily associated with mechanical wear and tribochemical interactions rather than widespread chemical degradation or aging-related phase transformation. Although zirconia is generally considered chemically inert [26], studies have indicated that prolonged exposure to acidic conditions can lead to its degradation [19,22,28]. However, such effects were not evident on the unworn surfaces in this study.

A previous study reported that acidic corrosion of zirconia may lead to surface accumulation of Al, Ca, K, and Fe oxides through alkali ion leaching [19]. In the present study, EDS analysis revealed only minor differences in these elements between groups. A reduction in surface yttrium content was observed in acid-exposed specimens compared to the distilled water group; however, given the semi-quantitative and surface-limited nature of EDS, this finding should not be interpreted as evidence of bulk yttria depletion. SEM imaging additionally revealed microcracks in acid-exposed specimens, and the uneven yttrium distribution within wear tracks (Figure 6) likely reflects localized surface compositional changes associated with tribochemical interactions rather than structural destabilization.

Conversely, wear did not differ significantly between speed and conventional sintering within any individual immersion medium, suggesting that the influence of the sintering condition on wear was considerably smaller than that of the immersion medium. This finding aligns with previous reports of comparable wear resistance between speed and conventional sintering [8,35]. Some studies have reported increased flexural strength with speed sintering due to its combination of high temperature and shorter sintering duration [32,55,56,57]. However, other studies indicate that speed sintering may weaken mechanical properties [33,34], while some report no significant reduction in mechanical performance despite the shortened sintering time [31,52,58,59]. These differences may be due to variations in furnace conditions or the use of zirconia materials not specifically designed for speed sintering. While previous studies have reported sintering-related microstructural differences in zirconia [32,60], the present findings suggest that these differences did not translate into significantly different wear outcomes under the tested conditions. Although speed-sintered zirconia showed slightly greater wear, this difference was not significant within any individual immersion medium. This minor variation may be associated with surface roughness or other microstructural factors such as residual porosity. Under the present experimental conditions, the effect of sintering condition on wear behavior appears limited.

Limitations of this study include the use of distilled water as a neutral immersion medium, which does not fully replicate the complex intraoral environment. In addition, only one commercial 5Y-ZP product was evaluated, and the findings may not be generalizable to other 5Y-ZP materials, which can differ in composition and processing. Biological factors such as salivary buffering capacity and acquired pellicle, which may influence zirconia wear behavior, were not considered in the present study [61]. Additionally, hydrochloric acid (HCl), which is relevant to GERD and bulimia patients, was excluded due to safety concerns. Zirconia antagonists were used to standardize the contact surface and minimize the variability associated with natural enamel. However, this may limit clinical relevance, as zirconia restorations most commonly oppose enamel, which differs from zirconia in hardness, microstructure and wear behavior; both the underlying wear mechanisms and the magnitude of wear may therefore differ under clinical conditions. Furthermore, the pH of the experimental media was not continuously monitored during the immersion period. Although freshly opened beverages were used, the pH may fluctuate over time, for instance due to the loss of carbonation in soft drinks, which might have influenced the wear behavior. In addition, surface characterization was limited to the micrometer scale. Surface roughness was reported as the two-dimensional profile parameter R_a_, and neither areal roughness parameters nor atomic force microscopy was applied; sub-micrometer topographical features could therefore not be quantified. As discussed above, the proposed IEP-related mechanism remains speculative and, because alkaline conditions were not tested, its confirmation will require further study across a broader pH range.

A simplified immersion model was intentionally adopted to isolate the effects of pH and acid type under standardized and reproducible laboratory conditions. Although artificial saliva better represents the oral environment, its inorganic and organic constituents may introduce additional variables that could obscure the specific influence of acidic environments on zirconia wear.

Future studies should therefore incorporate artificial saliva, thermocycling, and enamel or enamel-like antagonists; evaluate a broader pH range (including alkaline conditions) and longer acidic exposure; apply nanoscale and areal surface characterization, such as atomic force microscopy (AFM) or three-dimensional roughness mapping over larger areas, to characterize sintering-related microstructural differences; and test speed-sintered zirconia opposed by the same material, to better reproduce clinical conditions.

## 5. Conclusions

1. Within the limitations of this in vitro study of a single commercial 5Y-ZP material, acidic environments significantly influenced its wear behavior, with Coca-Cola exhibiting the lowest wear, followed by vinegar, whereas distilled water resulted in the highest wear. Although acidic conditions can contribute to zirconia corrosion, the observed wear patterns suggest that frictional wear and tribochemical processes played a greater role in overall material degradation. The wear under acidic conditions remained within clinically acceptable levels.
2. The sintering conditions had a limited influence on the wear resistance of 5Y-ZP zirconia. These findings suggest that speed sintering may represent a clinically acceptable and time-efficient alternative to conventional sintering, as its effect on wear was small compared with that of the immersion medium.
3. XRD analysis revealed no detectable monoclinic phase in any group, indicating that the observed wear differences occurred without any phase transformation measurable by the present method.

## Figures and Tables

**Table 1:** Mean, standard deviation (SD), and 95% confidence interval (CI) of the wear outcome in the tested groups.

Tested Groups		Volume Loss (mm^3^)			Mean Wear Depth (µm)			Surface Roughness (µm)		
Sintering Condition	Liquid Medium	Mean	SD	95% CI	Mean	SD	95% CI	Mean	SD	95% CI
Conventional	Distilled water	0.3211 ^a^	0.0546	0.2820–0.3602	53.408 ^c^	6.784	48.555–58.260	0.053 ^f^	0.012	0.044–0.062
Conventional	Coca-Cola	0.0027 ^b^	0.0012	0.0019–0.0036	1.870 ^e^	0.688	1.378–2.362	0.033 ^g^	0.006	0.028–0.037
Conventional	Vinegar	0.0360 ^b^	0.0116	0.0276–0.0443	11.575 ^d^	3.334	9.190–13.960	0.040 ^f,g^	0.011	0.032–0.048
Speed	Distilled water	0.3518 ^a^	0.1062	0.2758–0.4278	58.927 ^c^	13.199	49.485–68.369	0.052 ^f^	0.007	0.047–0.057
Speed	Coca-Cola	0.0049 ^b^	0.0027	0.0030–0.0069	2.899 ^e^	1.377	1.915–3.884	0.034 ^g^	0.010	0.027–0.041
Speed	Vinegar	0.0482 ^b^	0.0142	0.0381–0.0584	15.478 ^d^	2.584	13.630–17.326	0.042 ^f,g^	0.011	0.034–0.050

n = 10 specimens per group. Different superscript letters within the same column indicate statistically significant differences between groups (*p* < 0.05).

**Table 2:** Two-way ANOVA results (degrees of freedom, F-values, *p*-values, and effect sizes) for the effects of sintering condition and immersion medium on wear volume loss, mean wear depth, and surface roughness.

Outcome	Source	Df	*F*	*p*	Partial η^2^
Volume loss	Sintering	1	1.395	0.243	0.025
	Immersion medium	2	271.969	<0.001	0.910
	Sintering × medium	2	0.429	0.653	0.016
	Error	54	—	—	—
Mean wear depth	Sintering	1	4.544	0.038	0.078
	Immersion medium	2	402.250	<0.001	0.937
	Sintering × medium	2	0.645	0.529	0.023
	Error	54	—	—	—
Surface roughness	Sintering	1	0.132	0.717	0.002
	Immersion medium	2	19.688	<0.001	0.422
	Sintering × medium	2	0.078	0.925	0.003
	Error	54	—	—	—

Df, degrees of freedom; η^2^, partial eta squared (effect size). The error degrees of freedom were 54 for all outcomes.

**Table 3:** The surface elemental composition (at.%) of zirconia after a wear test under acid immersion. Values represent mean atomic percentages obtained from EDS measurements. For each group (n = 10), three specimens were selected, and four locations per specimen were analyzed. The reported values are averages of these measurements.

Elements	Conventional Sintering			Speed Sintering		
	Water	Coca-Cola	Vinegar	Water	Coca-Cola	Vinegar
Zr	19.4	21.3	18.2	19.8	20.8	13.2
C	68.2	55	58	61.5	61.7	73.4
Y	2.2	0.9	1.3	2.4	1.5	1
Al	0.2	1.6	3.2	0.2	0.7	0.9
Fe	0.2	0.2	0.1	0.3	0.1	0.4
Ca	0.08	0.1	0	0	0	0
K	0.1	0.8	0.2	0	0.2	0

Al, aluminum; Ca, calcium; C, carbon; Fe, iron; K, potassium; Y, yttrium; Zr, zirconium.

## Data Availability

The original contributions presented in this study are included in the article. Further inquiries can be directed to the corresponding author.
